# Glycemic Control Regimens in the Prevention of Surgical Site Infections: A Meta-Analysis of Randomized Clinical Trials

**DOI:** 10.3389/fsurg.2022.855409

**Published:** 2022-03-25

**Authors:** Jing Lai, Qihong Li, Ying He, Shiyue Zou, Xiaodong Bai, Sanjay Rastogi

**Affiliations:** ^1^Department of Nursing, The First People's Hospital of Longquanyi District, Chengdu, China; ^2^Department of Internal Medicine, Yantai Qishan Hospital, Yantai, China; ^3^Department of Science and Teaching, The First People's Hospital of Longquanyi District, Chengdu, China; ^4^Department of Endocrinology, The First People's Hospital of Longquanyi District, Chengdu, China; ^5^Department of Outpatient, China Medical University, Shenyang, China; ^6^Department of OMFS, Regional Dental College, Guwahati, India

**Keywords:** surgical site infections, glycemic control, laparoscopic surgeries, neurosurgeries, general surgery

## Abstract

**Background:**

Increased risk of surgical site infections (SSIs) caused by hyperglycemia makes it necessary to follow perioperative glucose lowering strategies to reduce postoperative complications. A meta-analysis was conducted to understand the efficacy of intensive vs. conventional blood glucose lowering regimens on the incidence of SSIs and hypoglycemia from various randomized controlled studies (RCTs).

**Materials and Methods:**

A systematic literature review was conducted using MEDLINE and Central databases for RCTs that involved intensive (lower blood glucose target levels) vs. conventional (higher blood glucose target levels) strategies in patients undergoing various types of surgeries. The primary outcomes were SSIs or postoperative wound infections. Hypoglycemia and mortality outcomes were also studied. A random-effects model was used to calculate the pooled risk ratio (RR), and subgroup analyses were performed.

**Results:**

A total of 29 RCTs were included in the meta-analysis with the information from 14,126 patients. A reduction in overall incidence of SSIs was found (RR 0.63, 0.50–0.80, *p* = 0.0002, *I*^2^= 56%). Subgroup analyses showed that intensive insulin regimens decreased the risk of SSIs in patients with diabetes, in cardiac and abdominal surgical procedures, and during the intraoperative and postoperative phases of surgery. However, the risk of hypoglycemia and mortality was increased in the intensive group compared to the conventional group.

**Conclusion:**

The results of the meta-analysis provide support for the use of intensive insulin regimens during the perioperative phase for decreasing the incidence of SSIs in certain patient populations and surgical categories.

## Introduction

Surgical site infections (SSIs) occur after an operative procedure and can range from superficial to deep wound infections. Global estimates of SSIs have ranged from 0.5 to 15% whereas studies in India have consistently shown higher rates from 23 to 38% (1). SSIs are a substantial cause of morbidity, prolonged hospitalization, hospital readmissions, and death and pose a considerable financial burden on healthcare systems (2, 3). Thus, the prevention and minimization of SSIs improve patient outcomes and reduce resource consumption (4, 5).

Strategies to reduce the risk of SSIs include interventions that can be delivered preoperatively, intraoperatively, or postoperatively. World Health Organization (WHO) and the Centers for Disease Control (CDC) have proposed guidelines recommending measures to prevent SSIs (6–8). Sterile procedures, maintaining patient homeostasis, wound closure interventions, and prophylactic antibiotics are commonly used to reduce the risk of SSIs (9).

Biological evidence demonstrates that diabetes can increase susceptibility to SSIs by compromising the immune system due to diminished leukocyte bactericidal activity and reduced chemotaxis and oxidative killing potential of neutrophils (10–12). Additionally, perioperative hyperglycemia or elevated blood glucose levels around the time of surgery are contributing factors to the risk of SSIs (13, 14). The perioperative period is the time surrounding a patient's surgical procedure and includes preoperative (before surgery), intraoperative (during surgery), and postoperative (after surgery) phases. Surgical stress causes the release of counter-regulatory catabolic hormones and inflammatory cytokines along with insulin tolerance and altered pancreatic β-cell function causing hyperinsulinemia and hyperglycemia (15–17). Based on the positive association of elevated blood glucose levels and SSIs, the CDC has recommended perioperative glycemic control and blood sugar levels <200 mg/dl in patients with and without diabetes for the prevention of SSIs (8). It is expected that intensive blood glucose lowering strategies will help in minimizing the risk of SSIs. However, low blood glucose levels are inherently associated with the risk of hypoglycemia, making it necessary to consider these adverse events.

Although several studies and meta-analysis on the benefits of perioperative glycemic control using intensive glucose control regimens (with stricter and lower blood glucose target levels) vs. conventional regimens (with higher blood glucose target levels) have been performed, there was no clear evidence in support of either of the treatments.

The objective of this paper is to synthesize current evidence from available randomized controlled trials evaluating the efficacy of intensive vs. conventional glucose control regimens in the prevention of SSIs in patients with and without diabetes. As lower blood glucose levels are associated with untoward outcomes like hypoglycemia, this meta-analysis also determines differences in hypoglycemia and mortality outcomes between intensive and conventional glucose control groups.

## Materials and Methods

We followed the guidelines of preferred reporting items for systematic reviews and meta-analyses (PRISMA) normative recommendations in this study with the registration number SU#/IRB/2020/9342.

### Search Strategy

A systematic literature search on MEDLINE (PubMed) and Cochrane Register of Controlled Trials (CENTRAL) was conducted in November 2021. No time limit was applied as several studies were published earlier than 1990. The following search terms were used in various combinations: surgical site infection, wound infection, SSI, postoperative, diabetes mellitus, hypoglycemia, insulin, blood glucose, and hypoglycemic agents. Additionally, a comprehensive list of search terms including Medical Subject Headings (MeSH) terms was applied. The titles and abstracts of studies that were potentially relevant were scanned, and the full text versions of the appropriate articles were read. Additional studies were identified by cross checking the reference lists of the relevant studies.

### Study Selection or Inclusion/Exclusion Criteria

Randomized controlled studies (RCTs) and prospective randomized studies that compared intensive insulin regimen with tighter, stricter blood glucose control vs. conventional regimens were included across various surgical categories. All studies reporting SSIs or wound infections as outcomes were included irrespective of the definition of SSIs used and even if they were not the primary outcomes. Exclusion criteria were non-randomized studies, quasi-experimental studies, retrospective studies, and cohort studies.

### Data Extraction and Quality Assessment

Following the identification of articles that met the inclusion criteria, data were extracted using a predefined data extraction form that included the following items: study author, publication year, surgery category, diabetes status of included patients, intervention, control, SSI data in each group, time of insulin administration, hypoglycemic events, and mortality.

The Cochrane Collaboration's risk of bias tool was used to assess the methodological quality of the included studies (18). This tool includes the following criteria: randomization, allocation concealment, blinding, and completeness of follow-up. The risk of bias for each item was graded as high, low, or unclear.

### Quantitative Data Synthesis

Meta-analysis was performed using Review Manager (RevMan, Version 5. Copenhagen: The Nordic Cochrane Center, The Cochrane Collaboration, 2020). Absolute numbers of participants in each study developing a wound infection or SSI and the total number of participants in each group (intervention and control group) were used to calculate the risk ratio (RR) and the 95% confidence interval (CI). Meta-analyses were done using a random-effects model (the Mantel–Haenszel method), and heterogeneity in the included studies was evaluated using *I*^2^ statistic, with small heterogeneity for *I*^2^ values of 25%, moderate heterogeneity for *I*^2^ values of 25–50%, and high heterogeneity for *I*^2^ values >50% (19). Forest plots were constructed, and *p* < 0.05 was statistically significant. Subgroup analyses were also performed according to target blood glucose levels of intervention group, diabetes status of patients, timing of insulin administration, surgery type, and types of SSI.

Publication bias was assessed by a funnel plot in which the log RR for each study was plotted against its SE.

## Results

### Identification of Studies

**Figure 1:**
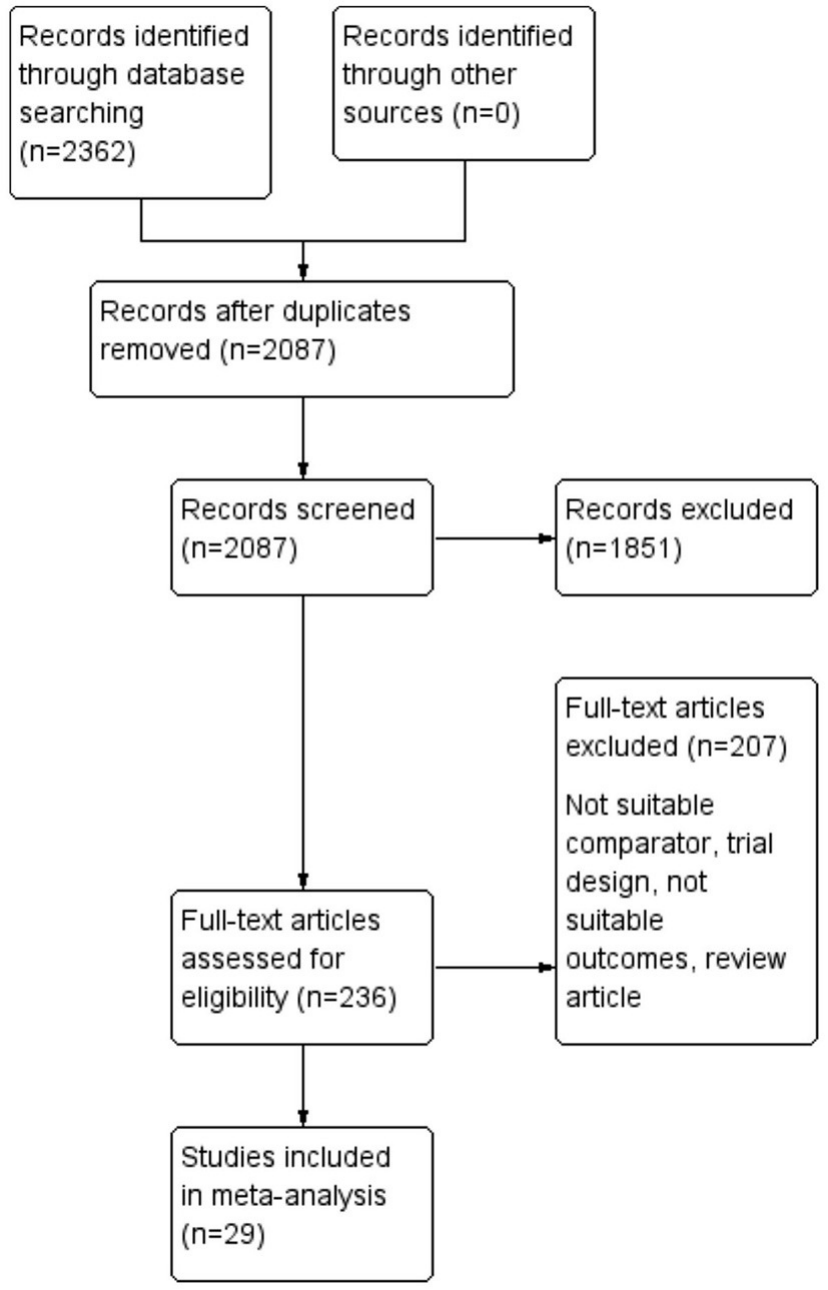
A flow chart for the identification and inclusion of studies in the meta-analysis according to Preferred Reporting Items for Systematic and Meta-Analyses (PRISMA).

A total of 2,362 records were identified by database searching of which 2,087 were screened by the title and abstract. Irrelevant records were removed (*n* = 1,851), and 236 RCTs were assessed for eligibility. However, 207 RCTs were excluded due to reasons such as inappropriate comparator groups, wound infections not reported as outcomes, an inappropriate trial design, and the lack of data. The process of selection is shown in Figure 1.

### Study Characteristics

In total, 29 RCTs totaling 14,126 participants met the inclusion criteria (intensive intervention group: 7,351 participants and control group: 6,775 participants). These RCTs involved a comparison of intensive blood glucose control to conventional blood glucose control or standard treatment regimens across various surgical categories in the perioperative period. All studies were randomized controlled trials or prospective randomized studies with sample sizes ranging from 26 to 6,025 participants. The studies included male and female participants undergoing various surgical procedures both with and without diabetes or mixed populations. All studies utilized insulin infusion in the intensive treatment group whereas insulin infusion and sliding-scale subcutaneous insulin administration were used in the conventional treatment group. SSIs were defined according to different criteria in the studies (Tables 1, 2). In the intensive treatment groups, blood glucose levels were mainly targeted between 80 and 120 mg/dl and liberal blood glucose values of <250 mg/dl were used in the conventional group.

**Table 1:** Demographic characteristics of the included studies.

**References**	**Surgery type**	**Population (diabetic and non-diabetics)**
Abdelmalak et al. (20)	Non-cardiac	Mixed
Agus et al. (21)	Cardiac	Non-diabetics
Akabori et al. (22)	Pancreaticduodectomy	Diabetics
Albacker et al. (23)	CABG	Mixed
Albacker et al. (24)	CABG	Mixed
Bilotta et al. (25)	Aneurysm	Mixed
Bilotta et al. (26)	Brain surgery	Mixed
Bilotta et al. (27)	Neurosurgery	Mixed
Cao et al. (28)	Open elective gastrectomy	Diabetics
Cao et al. (29)	Gastrectomy	Non-diabetics
Chan et al. (30)	Cardiac	Mixed
De La Rosa et al. (31)	ICU	Mixed
Desai et al. (32)	CABG	Mixed
Emam et al. (33)	Cardiac	Diabetics
Furnary et al. (34)	Cardiac	Diabetics
Gandhi et al. (35)	Cardiac	Mixed
Grey et al. (36)	Critical	Mixed
Kirdemir et al. (37)	CABG	Diabetics
Lazar et al. (38)	CABG	Diabetics
Lazar et al. (39)	CABG	Diabetics
Li et al. (40)	CABG	Diabetics
NICE-SUGAR Study Investigators (41)	ICU	Mixed
Okabayashi et al. (42)	Pancreatic	Mixed
Rassias et al. (43)	Cardiac	Diabetics
Subramaniam et al. (44)	Vascular	Mixed
Tohya et al. (45)	Oral and maxillofacial surgery	Mixed
Wahby et al. (46)	CABG	Diabetics
Yuan et al. (47)	Gastrectomy	Diabetics
Zheng et al. (48)	Cardiac	Non-diabetics

**Table 2:** Study characteristics of the included studies.

**References**	**Intervention**	**Control**	**SSI definition**
Abdelmalak et al. (20)	IV insulin infusion (target BG: 80–110 mg/dl)	IV insulin infusion (target BG: 180–200 mg/dl)	Deep and organ space
Agus et al. (21)	IV insulin infusion (target BG: 80–100 mg/dl)	Standard care	CDC criteria
Akabori et al. (22)	Artificial pancreas control (target BG: 80–100 mg/dl)	Insulin infusion (target BG < 180 mg/dl)	CDC criteria
Albacker et al. (23)	IV insulin infusion (target BG: 70–110 mg/dl)	Sliding scale SC insulin (target BG < 180 mg/dl)	Superficial
Albacker et al. (24)	IV insulin infusion with 20% dextrose (target BG: 70–110 mg/dl)	Sliding scale SC insulin (target BG < 180 mg/dl)	Superficial
Bilotta et al. (25)	IV insulin infusion (target BG: 80–120 mg/dl)	IV insulin infusion (target BG: 80–200 mg/dl)	NNIS definition
Bilotta et al. (26)	Insulin infusion (target BG: 80–120 mg/dl)	Insulin infusion (target BG < 200 mg/dl)	NNIS definition
Bilotta et al. (27)	Insulin infusion (targer BG: 80–110 mg/dl)	Insulin infusion (target BG < 214 mg/dl)	NNIS definition
Cao et al. (28)	IV insulin infusion (target BG: 800–100 mg/dl)	IV insulin infusion (target BG < 200 mg/dl)	CDC criteria
Cao et al. (29)	IV insulin infusion (target BG: 800–100 mg/dl)	IV insulin infusion (target BG < 200 mg/dl)	CDC criteria
Chan et al. (30)	IV insulin infusion (target BG: 80–130 mg/dl)	IV insulin infusion (target BG: 160–200 mg/dl)	Not specified
De La Rosa et al. (31)	Insulin infusion (target BG: 80–110 mg/dl)	Insulin infusion (target BG: 180–200 mg/dl)	CDC criteria
Desai et al. (32)	Target BG: 90–100 mg/dl	Target BG: 121–180 mg/dl	Deep sternal wound infection
Emam et al. (33)	IV insulin infusion (target BG: 100–150 mg/dl)	Sliding scale SC insulin (target BG < 200 mg/dl)	Superficial and deep
Furnary et al. (34)	Insulin infusion (target BG: 150–200 mg/dl)	Sliding scale SC insulin (target BG < 200 mg/dl)	Deep sternal wound infection
Gandhi et al. (35)	IV insulin infusion (target BG: 80–100 mg/dl)	IV insulin infusion (target BG < 200 mg/dl)	Deep sternal infection
Grey et al. (36)	IV insulin infusion (target BG: 80–120 mg/dl)	IV insulin infusion (target BG: 180–220 mg/dl)	Not specified
Kirdemir et al. (37)	IV insulin infusion (target BG: 100–150 mg/dl)	Sliding scale SC insulin (target BG < 200 mg/dl)	Sternal wound infection
Lazar et al. (38)	Glucose–insulin–potassium solution (target BG: 125–200 mg/dl)	Standard treatment (target BG < 250 mg/dl)	Wound
Lazar et al. (39)	IV insulin infusion (target BG: 90–120 mg/dl)	IV insulin infusion (target BG: 120–180 mg/dl)	Sternal wound infection
Li et al. (40)	Continuous insulin infusion	SC insulin (target BG: 150–200 mg/dl)	Sternal wound infection
NICE-SUGAR Study Investigators (41)	Insulin infusion (target BG: 81–108 mg/dl)	Insulin infusion (target BG < 180 mg/dl)	Positive blood culture
Okabayashi et al. (42)	IV insulin infusion (target BG: 80–110 mg/dl)	IV insulin infusion (target BG: 140–180 mg/dl)	CDC and NNIS definition
Rassias et al. (43)	Aggressive insulin therapy	Standard insulin therapy	Septic mediastinitis
Subramaniam et al. (44)	Insulin infusion (target BG: 100–150 mg/dl)	Standard intermittent sliding–scale insulin bolus (target BG < 150 mg/dl)	Not specified
Tohya et al. (45)	Insulin infusion (target BG: 80–120 mg/dl)	Ringer's lactate solution (target BG < 180 mg/dl)	SSI (MRSA-positive)
Wahby et al. (46)	Insulin infusion (target BG: 110–149 mg/dl)	Target BG: 150–180 mg/dl	Sternal wound infection
Yuan et al. (47)	Insulin infusion (target BG: 80–110 mg/dl)	Intermittent bolus insulin (target BG < 200 mg/dl)	Not specified
Zheng et al. (48)	IV insulin infusion (target BG: 70–110 mg/dl)	Standard care, no control of BG	Deep sternal wound infection

*SSI, surgical site infection; BG, blood glucose; CDC, Centers for Disease Control and Prevention; NNIS, National nosocomial infections surveillance system*.

### Types of Surgery and Timing of Administration

Most studies were conducted on participants undergoing cardiac surgery (*n* = 15), neurosurgery (*n* = 3), and abdominal procedures (*n* = 4). In 5 studies, insulin was administered in the intraoperative phase, intraoperative and postoperative insulin administration was carried out in 15 studies, and in 7 studies insulin was administered postoperatively.

### Bias Assessment

**Figure 2:**
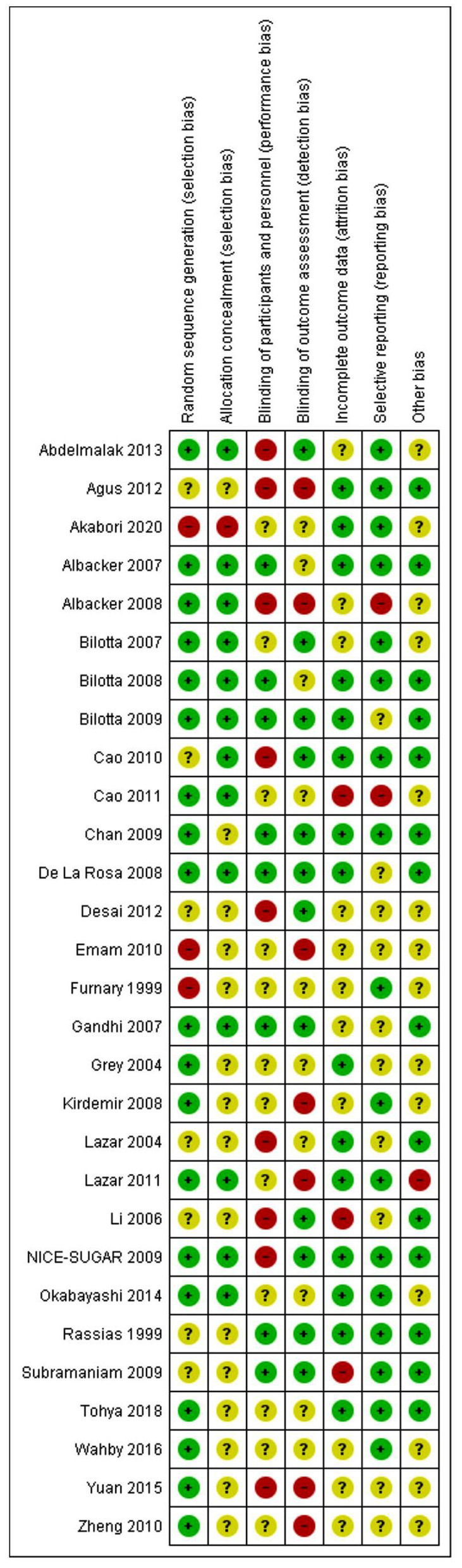
Risk of bias summary for trials included in the meta-analysis (*n* = 29).

The results of the risk of bias evaluation are shown in Figure 2. Overall, there was a moderate to high risk of bias due to an unclear or a high risk related to randomization, blinding, and selective reporting domains.

**Figure 3:**
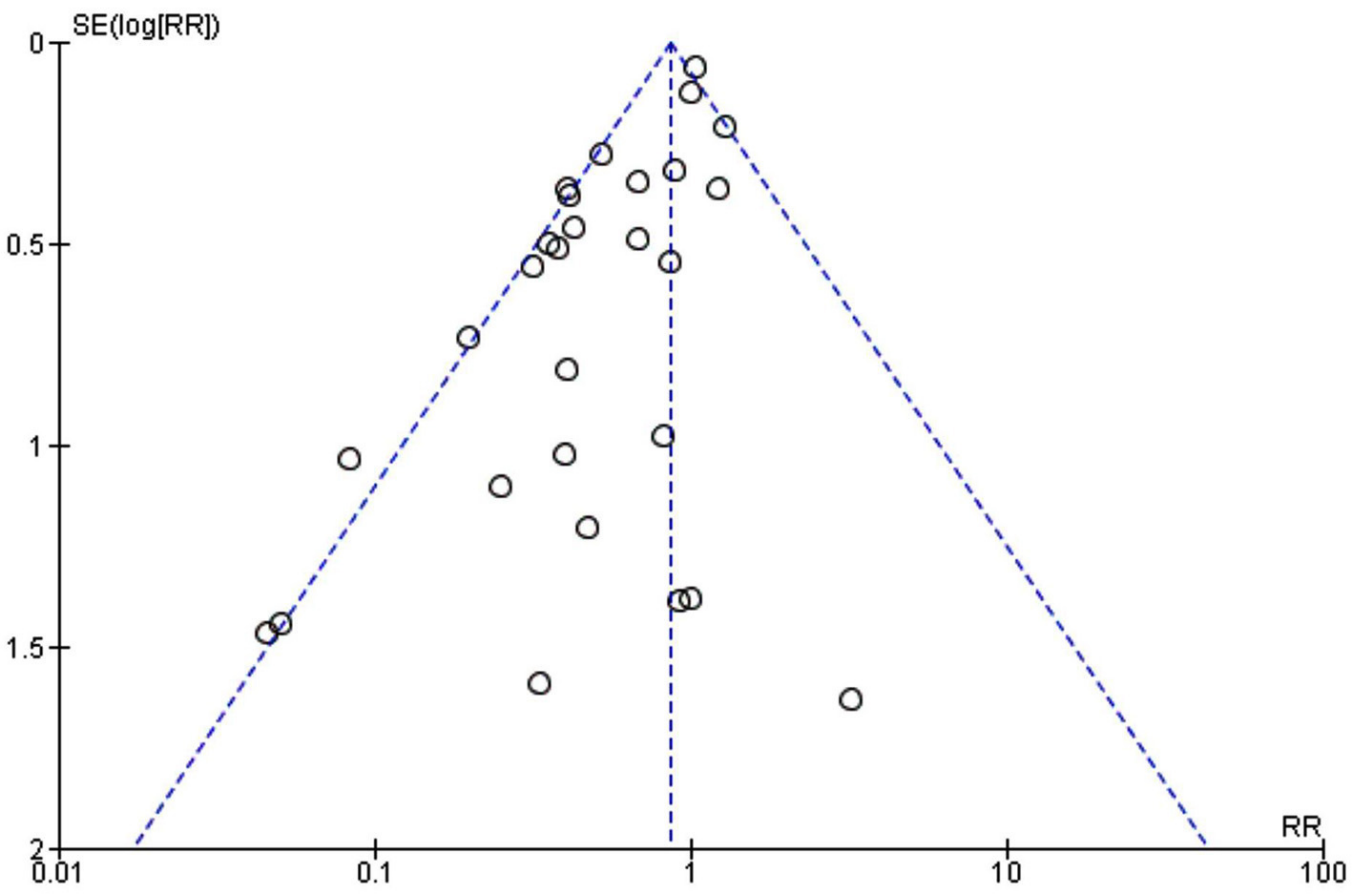
A funnel plot to assess publication bias in meta-analysis with the surgical site infection (SSI) outcome.

The funnel plot was asymmetrical (Figure 3), indicating the possibility of publication bias.

### Surgical Site or Wound Infection Rates

The incidence of a SSI or wound infection in the included studies is shown in Table 3. The incidence of SSIs ranged from 0 to 32.3% in the intensive intervention group and from 0 to 66.7% in the conventional treatment group. The overall incidence of SSIs was 8.6% in the intensive intervention group and 10.7% in the conventional treatment group. Table 4 shows data for the types of SSI reported in the included studies.

**Table 3:** Surgical site infection (SSI) rates in included studies.

**References**	**SSI rate (%)**	
	**Intervention**	**Control**
Abdelmalak et al. (20)	8.7	9.7
Agus et al. (21)	3.3	2.7
Akabori et al. (22)	28.6	66.7
Albacker et al. (23)	4.5	4.5
Albacker et al. (24)	3.7	4.0
Bilotta et al. (25)	2.5	5.3
Bilotta et al. (26)	4.2	10.2
Bilotta et al. (27)	5.4	7.9
Cao et al. (28)	4.3	13.8
Cao et al. (29)	4.0	10.6
Chan et al. (30)	11.1	16.4
De La Rosa et al. (31)	32.3	32.8
Desai et al. (32)	1.1	0
Emam et al. (33)	0	12.5
Furnary et al. (34)	0.80	2.0
Gandhi et al. (35)	3.2	3.8
Grey et al. (36)	5.9	29.4
Kirdemir et al. (37)	1.0	12.0
Lazar et al. (38)	0	13.0
Lazar et al. (39)	0	0
Li et al. (40)	3.9	4.8
NICE-SUGAR Study Investigators (41)	12.8	12.4
Okabayashi et al. (42)	4.1	9.8
Rassias et al. (43)	0	7.7
Subramaniam et al. (44)	30.7	23.8
Tohya et al. (45)	10	25
Wahby et al. (46)	20.9	39.7
Yuan et al. (47)	4.7	13.2
Zheng et al. (48)	2.0	8.0

**Table 4:** Proportion of different type of SSI in the included studies.

**References**	**Superficial SSI**	**Deep**	**Organ/space SSI**	**Mixed**	**Skin dehiscence**
Abdelmalak et al. (20)	NA	*I: 17/196 *C: 18/185		NA	NA
Agus et al. (21)	NA	NA		NA	NA
Akabori et al. (22)	I: 1/14 C: 1/15	NA	I: 3/14 C: 9/15	NA	NA
Albacker et al. (23)	I: 1/22 C: 1/22	NA	NA	NA	NA
Albacker et al. (24)	I: 1/27 C: 1/25	NA	NA	NA	NA
Bilotta et al. (25)	NA	NA	NA	NA	NA
Bilotta et al. (26)	NA	NA	NA	NA	NA
Bilotta et al. (27)	NA	NA	NA	NA	NA
Cao et al. (28)	NA	NA	NA	NA	NA
Cao et al. (29)	NA	NA	NA	NA	NA
Chan et al. (30)	NA	NA	NA	NA	NA
De La Rosa et al. (31)	NA	NA	NA	NA	NA
Desai et al. (32)	NA	I: 1/91 C: 0/98	NA	NA	NA
Emam et al. (33)	I: 0/80 C: 3/40	I: 0/80 C: 2/40	NA	NA	NA
Furnary et al. (34)	NA	NA	NA	NA	NA
Gandhi et al. (35)	NA	I: 6/185 C: 7/186	NA	NA	NA
Grey et al. (36)	NA	NA	NA	NA	NA
Kirdemir et al. (37)	NA	NA	NA	NA	NA
Lazar et al. (38)	NA	NA	NA	NA	NA
Lazar et al. (39)	NA	NA	NA	NA	NA
Li et al. (40)	NA	NA	NA	I: 2/51 C: 2/42	NA
NICE-SUGAR Study Investigators (41)	NA	NA	NA	NA	NA
Okabayashi et al. (42)	NA	NA	NA	NA	NA
Rassias et al. (43)	NA	NA	NA	NA	NA
Subramaniam et al. (44)	NA	NA	NA	NA	NA
Tohya et al. (45)	NA	NA	NA	NA	NA
Wahby et al. (46)	NA	NA	NA	NA	NA
Yuan et al. (47)	NA	NA	NA	NA	NA
Zheng et al. (48)	NA	NA	NA	NA	NA

*I, intensive regimen; C, conventional regimen*.

### Meta-Analysis Results

**Figure 4:**
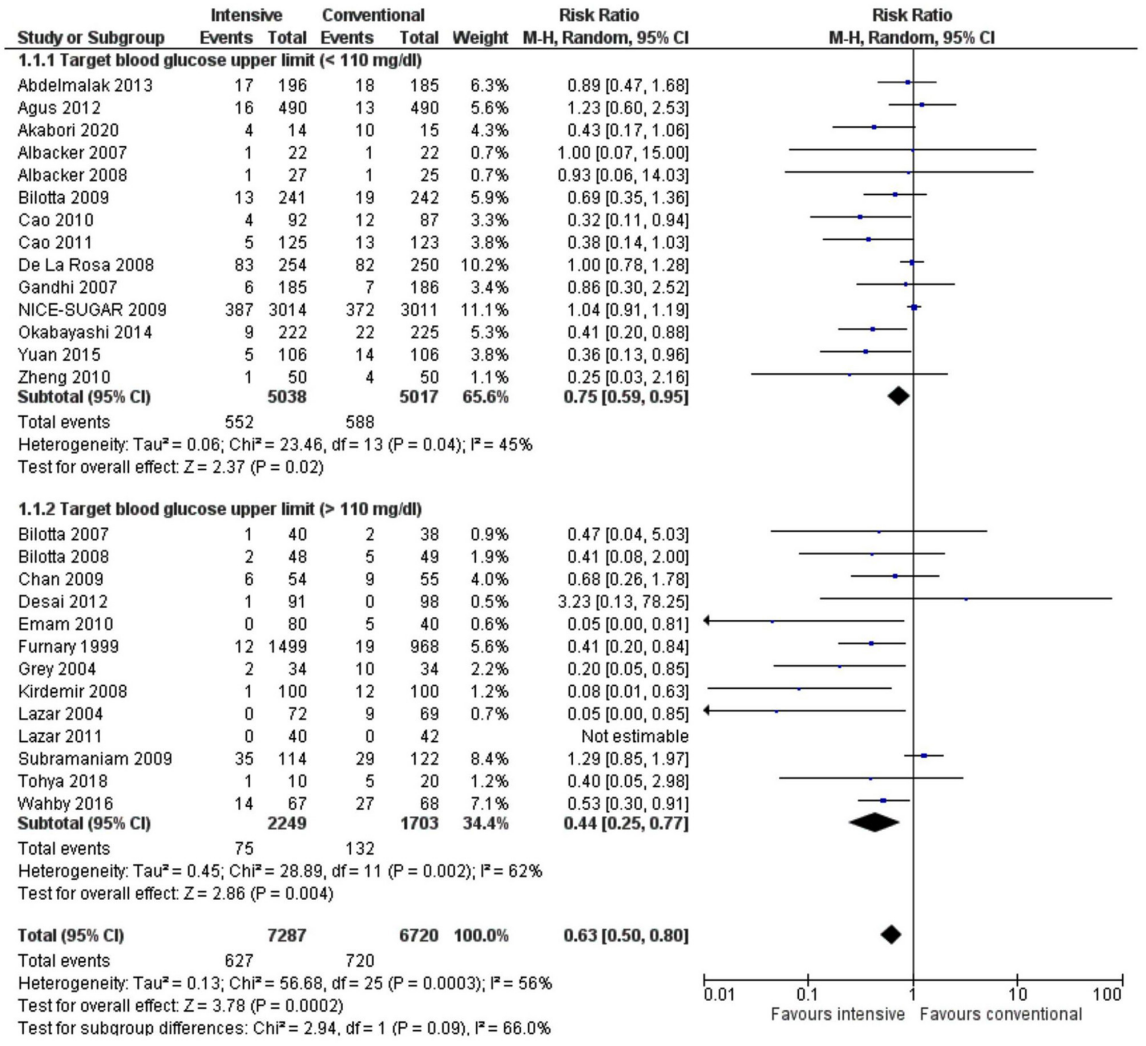
A forest plot for a subgroup analysis of target blood glucose level in the intervention group in studies using a random-effects model. Risk ratios (RRs) and 95% CIs are shown.

In addition, there was no significant difference between the subgroups indicating that the intensive regimen was preferred irrespective of the target blood glucose levels. Moderate to high heterogeneity could be attributed to the diabetes status of patients or the types of surgery (Figure 4).

**Figure 5:**
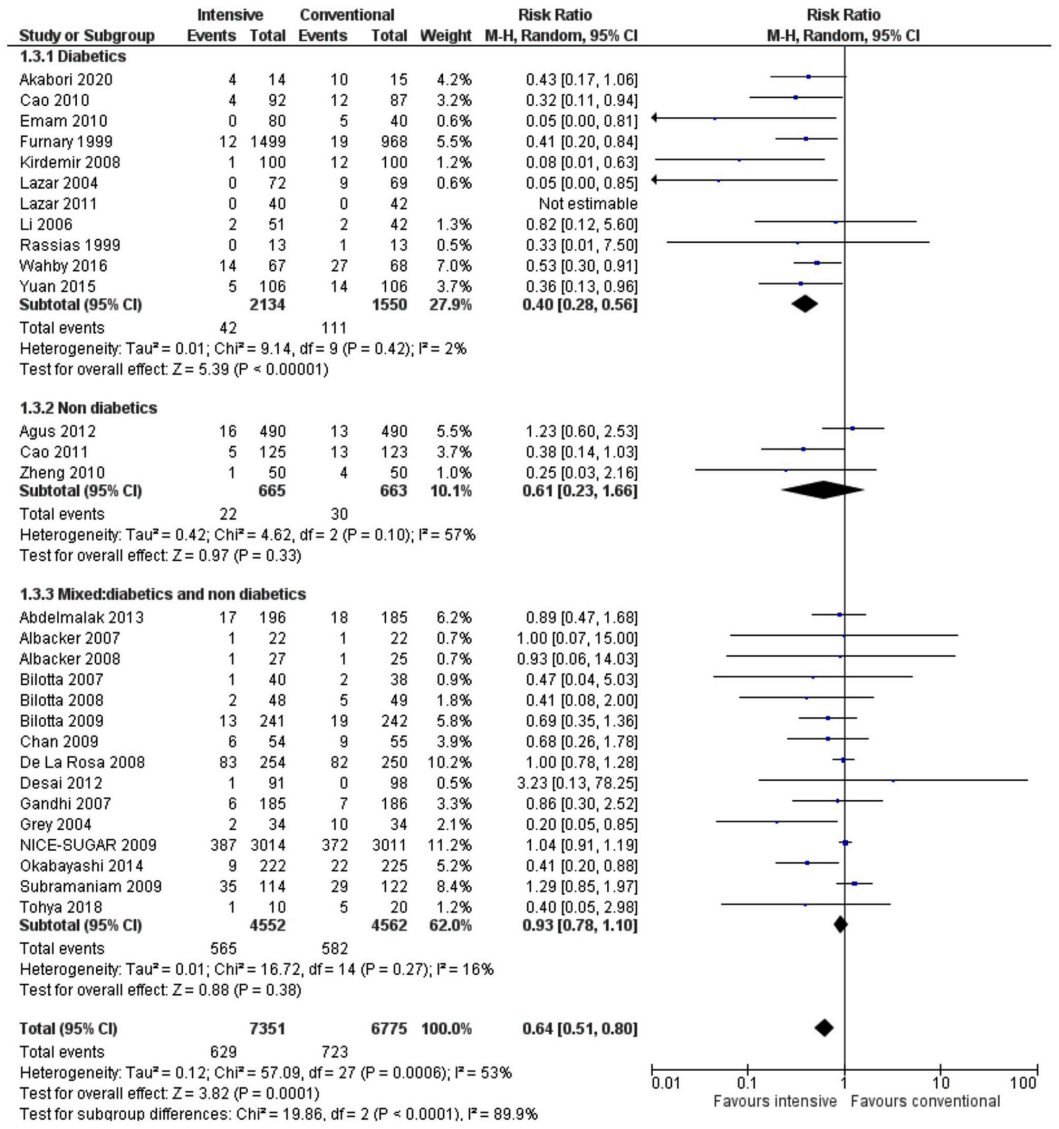
A forest plot for a subgroup analysis depending on the patient diabetes status in studies using a random-effects model. RRs and 95% CIs are shown.

Stratification of the results by the patient population (diabetic, non-diabetic, or mixed) showed a significant decrease in the incidence of SSIs in favor of the intensive treatment group for the diabetic population only (RR 0.40, 0.28–0.56, *p* < 0.00001, *I*^2^ = 2%). In contrast, there was no significant difference in the SSI rate for studies, including non-diabetics (RR 0.61, 0.23–1.66, *p* = 0.33, *I*^2^ = 57%) or studies with both diabetic and non-diabetic patients (RR 0.93, 0.78–1.10, *p* = 0.38, *I*^2^= 16%) (Figure 5). The test for subgroup differences indicated a statistically significant subgroup difference (*p* < 0.0001), indicating that patient diabetic status does influence the response to the intensive glycemic control treatment. High heterogeneity (*I*^2^ = 57%) in the nondiabetic subgroup could be due to a low number of studies in this population and their inclusion in the analysis (*n* = 3).

**Figure 6:**
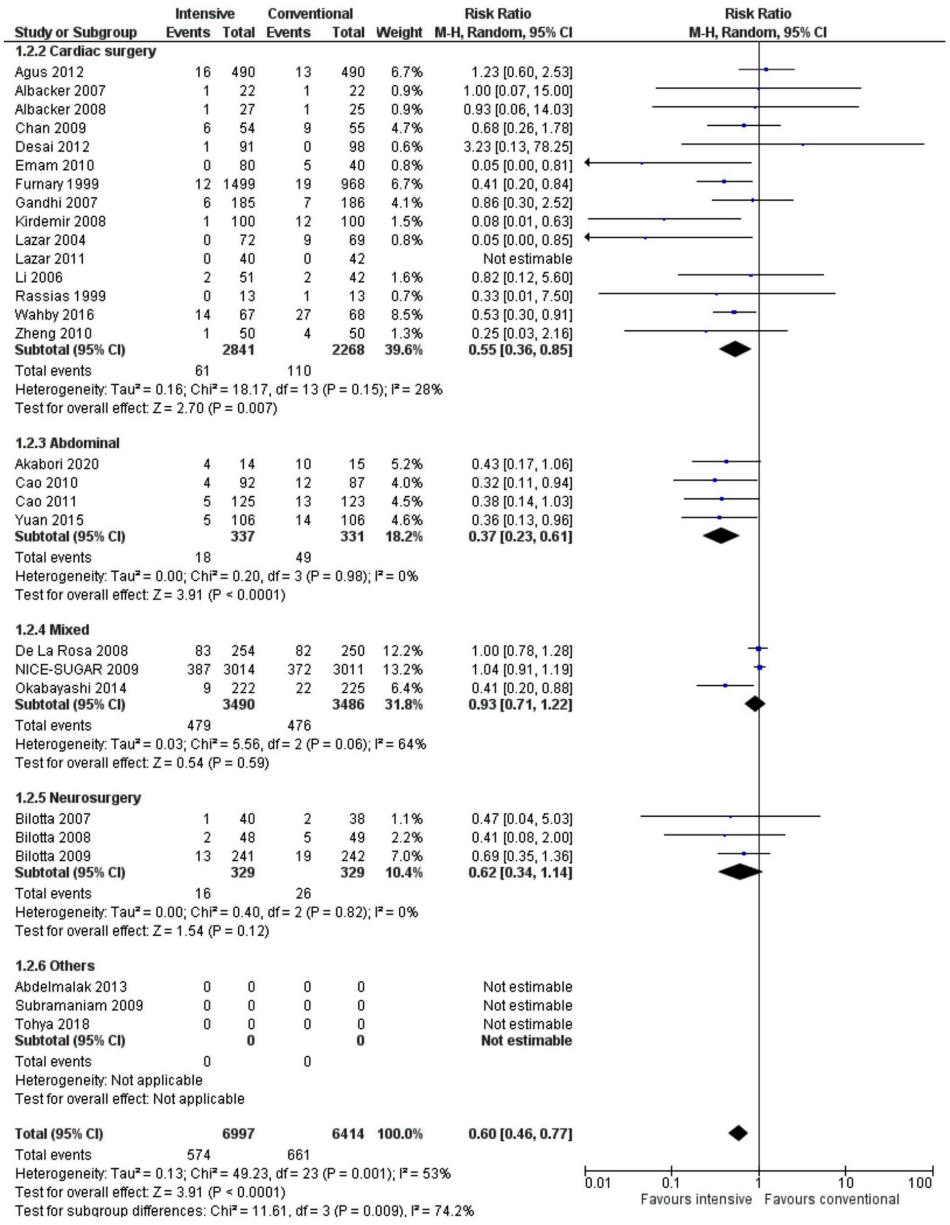
A forest plot for a subgroup analysis according to surgery type in studies using a random-effects model. RRs and 95% CIs are shown.

A subgroup analysis showed that a decrease in the incidence of SSIs was statistically significant following intensive insulin treatment in cardiac surgery (RR 0.55, 0.36–0.85, *p* = 0.007, *I*^2^ = 28%) and abdominal surgery (RR 0.37, 0.23–0.61, *p* < 0.0001, *I*^2^ = 0%) but not in neurosurgery (RR 0.62, 0.34–1.14, *p* = 0.12, *I*^2^ = 0%). Furthermore, there was a significant influence of the type of surgery (*p* = 0.009) on the effect of the intensive insulin regimen (Figure 6).

**Figure 7:**
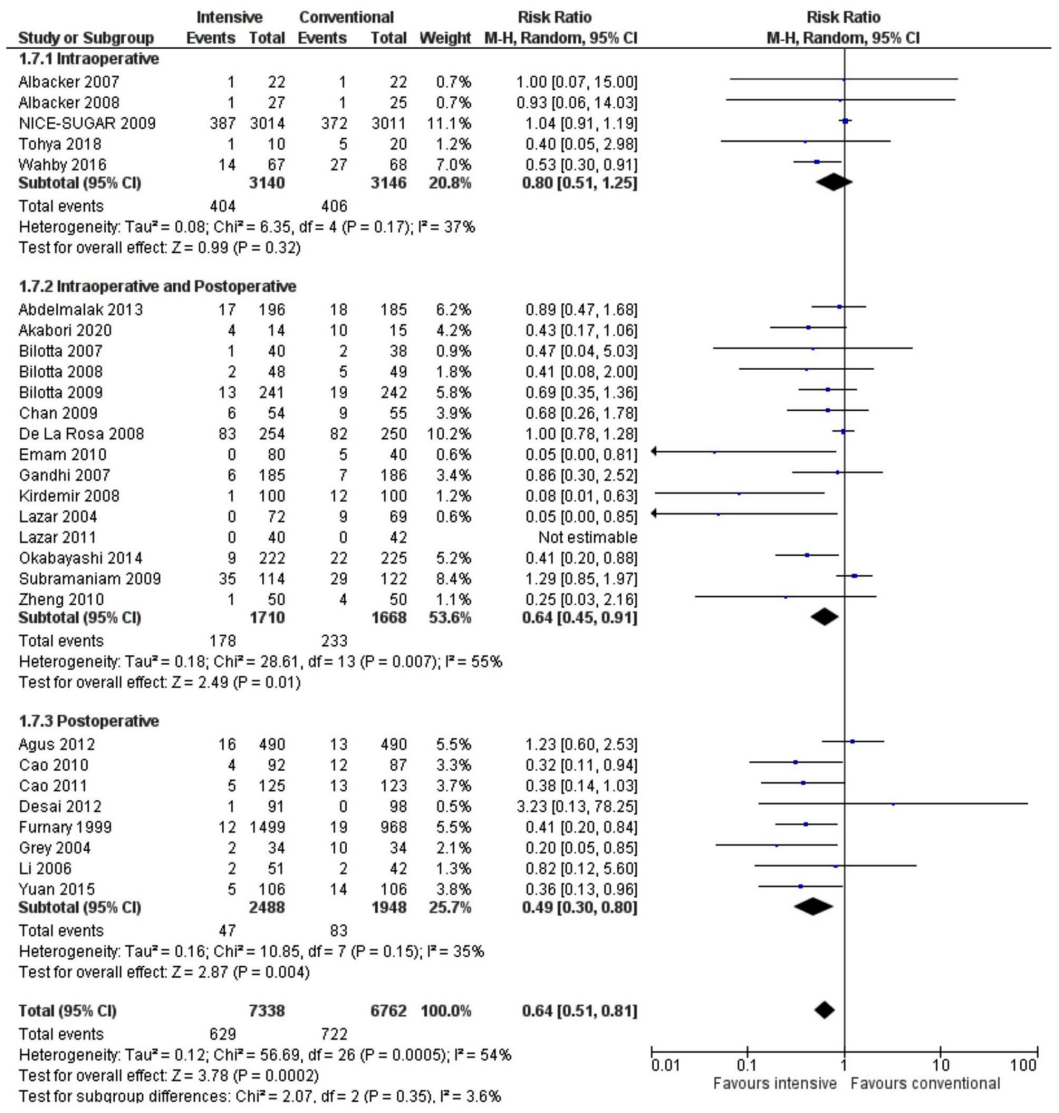
A forest plot for a subgroup analysis according to timing of insulin administration in studies using a random-effects model. RRs and 95% CIs are shown.

Intraoperative and postoperative insulin administration or only postoperative administration was associated with a significantly lower SSI risk (RR 0.64, 0.45–0.91, *p* = 0.01, *I*^2^ = 55% and RR 0.49, 0.30–0.80, *p* = 0.004, *I*^2^ = 35%) compared to when intensive treatment was done only during the intraoperative phase (RR 0.80, 0.51–1.25, *p* = 0.32, *I*^2^ = 37%) (Figure 7).

**Figure 8:**
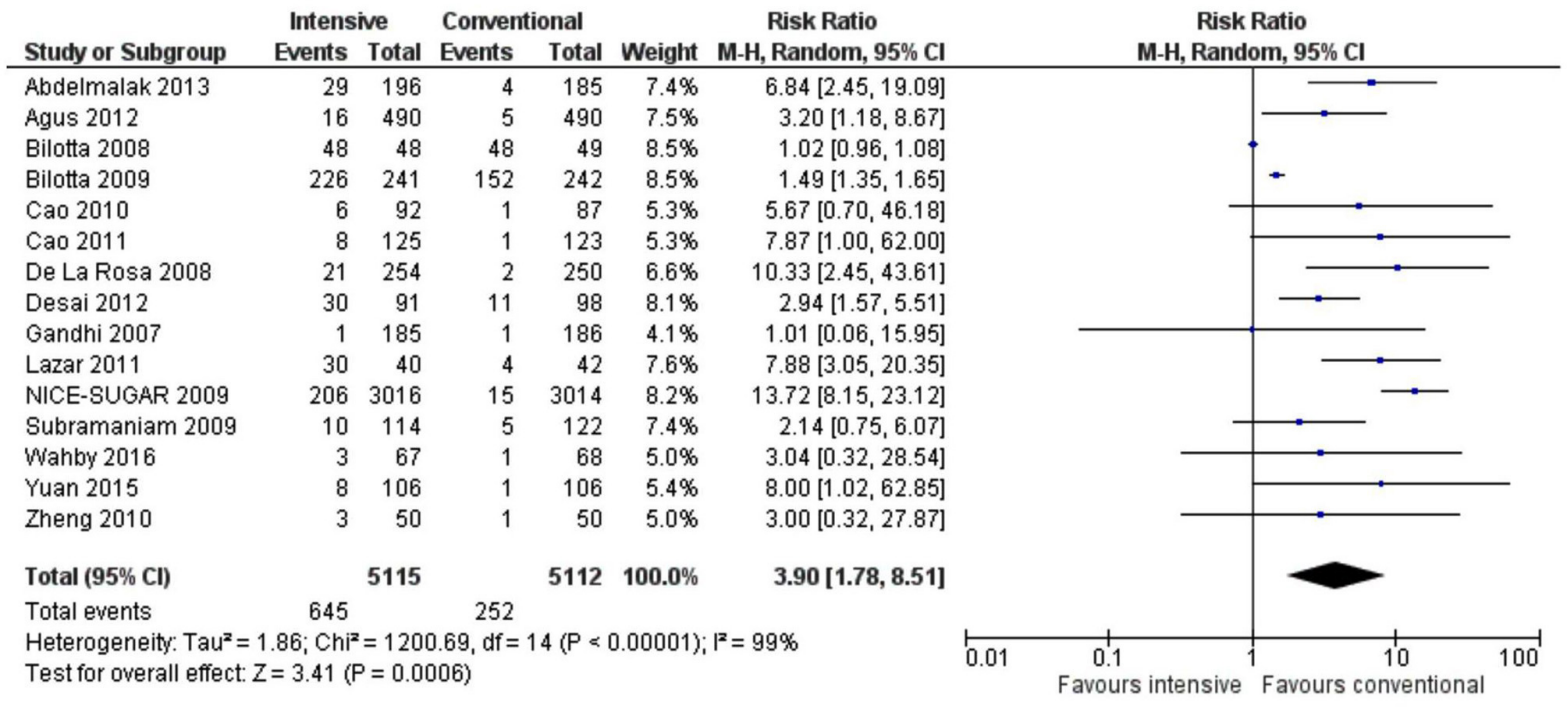
A forest plot of hypoglycemic events for studies using a random-effects model. RRs and 95% CIs are shown.

**Figure 9:**
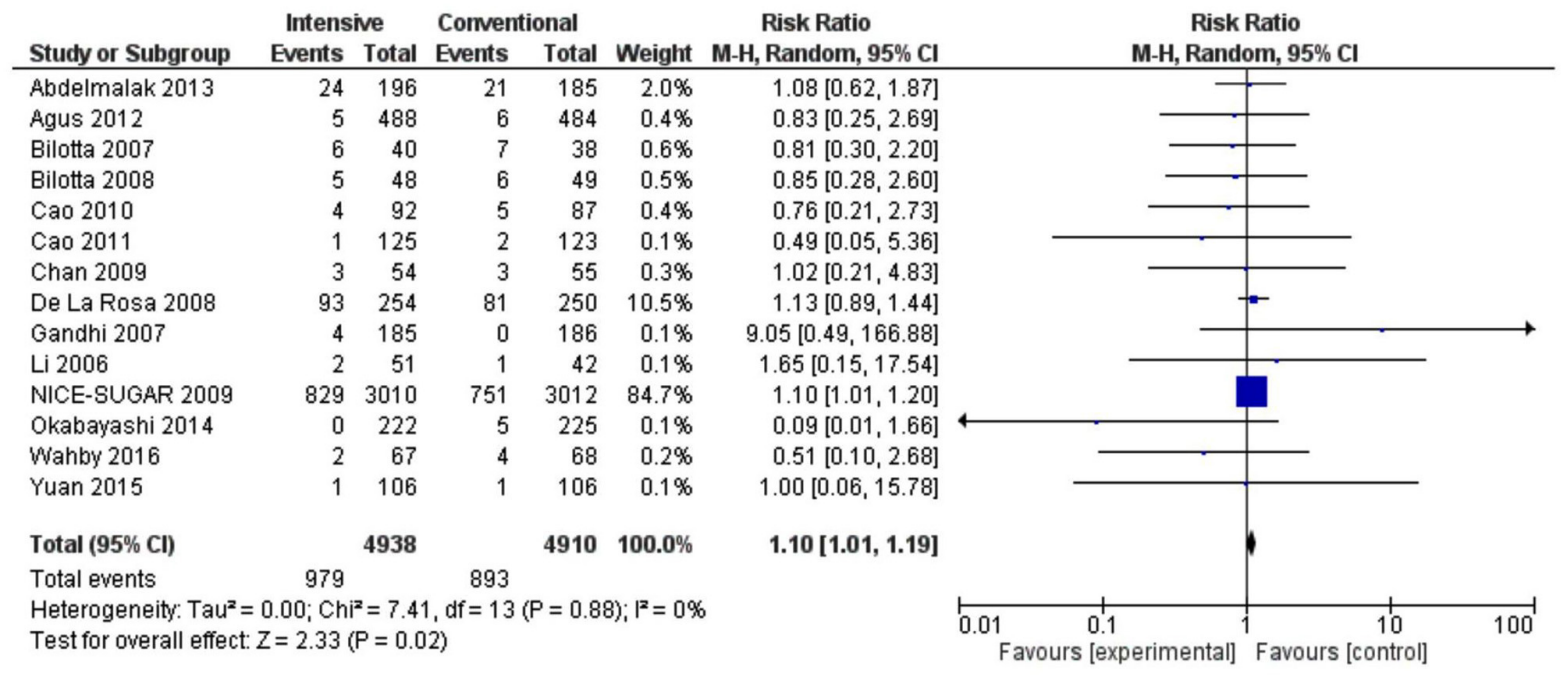
Forest plot of mortality outcome for studies using a random-effects model. RRs and 95% CIs are shown. Experimental: intensive, control: conventional.

The risk of hypoglycemia and mortality was significantly higher in the intensive treatment compared to the conventional group (RR 3.90, 1.78–8.51, *p* = 0.0006, *I*^2^ = 99% and RR 1.10, 1.01–1.19, *p* = 0.02, *I*^2^ = 0%) (Figures 8, 9). High heterogeneity for the hypoglycemia outcome could be due to various surgical procedures and mixed patient populations as well as an inconsistent definition for hypoglycemia.

There was no significant difference in the incidence of superficial or deep SSIs between intensive and conventional groups (superficial SSI RR 0.56, 0.14–2.22, *p* = 0.41, *I*^2^ = 0% and deep SSI RR 0.86, 0.51–1.45, *p* = 0.45, *I*^2^ = 0%).

## Discussion

The present study provides current and valuable information on the efficacy of intensive blood glucose lowering strategies in decreasing the incidence of SSIs across various surgical categories and in different patient populations with regard to the diabetes status. In this meta-analysis, most studies utilized intensive insulin treatment for targeting blood glucose levels 70–120 mg/dl in the intraoperative and postoperative, and only postoperative phases of surgery.

Unlike a previous meta-analysis on a similar topic, the current meta-analysis attempts to categorize different subgroups and determine the efficacy of intensive insulin treatment by a subgroup, thus attempting to determine where tight glucose control is most likely to be beneficial. The utilization of intensive glucose control protocols in diabetic patient populations, cardiac, and abdominal surgical procedures, and during the intraoperative and postoperative and only the postoperative phase of surgery were shown to be associated with a decreased risk of SSIs compared to when conventional strategies were used. Furthermore, a decrease in the incidence of SSIs was observed irrespective of whether target blood glucose levels during intensive treatment were <110 or >110 mg/dl. Kao et al. (13) concluded that studies comparing intensive and conventional glycemic control regimens could not be meta-analyzed due to heterogeneity in patient populations, the type of surgery, mortality risk, timing of administration, the route of administration, and target blood glucose levels. A recent meta-analysis in 2017 by de Vries et al. included 15 RCTs and showed that stricter blood glucose target levels of <150 mg/dl using reduced SSIs with an inherent risk of hypoglycemic events (49). This meta-analysis includes more studies and provides pooled effect estimates for hypoglycemic events and adverse events such as mortality.

Despite the beneficial effects of lowering SSI rates, the current meta-analysis showed a higher risk of mortality caused by intensive insulin therapy. However, it is important to note most of this data came from the Normoglycemia in Intensive Care Evaluation-Survival Using Glucose Algorithm Regulation (NICE-SUGAR) study which included critically ill patients and in which the blood glucose target levels in the intensive insulin group were comparatively lower than other studies (81–108 mg/dl) (41). Similar results were seen in the study by Gandhi (35) wherein the achieved blood glucose levels were much lower. However, increased hypoglycemic events were seen in almost all studies that reported hypoglycemia as an outcome. High heterogeneity seen in the meta-analysis of the results for hypoglycemia can be attributed to differences in definitions of hypoglycemia used between the included trials, which were either the number of patients experiencing at least one hypoglycemic episode or the percentage of glucose measurements below a cut-off value. Increased monitoring of glucose levels in the intensive groups vs. conventional groups could have also resulted in measurement bias and increase in hypoglycemic events. Therefore, these results should be interpreted with caution and may not provide an exact estimate. Furthermore, hypoglycemic episodes in most studies were asymptomatic and the reduction in SSIs probably outweighs the risk of hypoglycemia, thereby supporting the use of intensive treatment. With regard to oral surgery, Tohya et al. (45) investigated the effects of intraoperative glycemic control by glucose–insulin infusion to achieve a target blood glucose level of 80–120 mg/dl in patients with major oral or maxillofacial surgeries compared to the infusion of Ringer's lactate solution alone. This study did not find a significant difference in the incidence of SSIs between the groups (*p* = 0.31) but is limited by a small sample size (*n* = 30) (45).

Although there was no significant difference between the rates of SSIs in intensive and conventional glucose control groups when stratified by the types of SSI, the small number of studies used to obtain the results should be considered during interpretation. Additionally, the types of SSI (superficial or deep) was reported only in case of cardiac surgeries. Most studies reported the incidence of overall SSIs or wound infections.

## Limitations

Although this meta-analysis provides insights into which cases intensive insulin treatment may be beneficial, there are some limitations. The definition of SSIs and follow-up times differed among studies, which can potentially affect the results. Several other studies were found during the literature search process that involved a comparison of intensive with conventional glucose control regimens, which reported infections as outcomes. However, the lack of data on SSIs or wound infections resulted in these studies being excluded from the analysis. Details on postoperative nutritional protocols and antibiotic or other concomitant medication use were not reported in most studies, which can confound the results. Most studies included in the meta-analysis were performed in patients undergoing cardiac surgery and those reporting ICU stay, which limits generalizability of the results to a wider patient population. In most cases, the achieved blood glucose levels are associated with the outcomes of SSIs and hypoglycemia and not the target level or route of administration, which makes it essential to report actual achieved levels in all the studies. Another important aspect that needs to be considered when evaluating perioperative glucose control for the reduction of SSIs is the safety and feasibility of administering insulin as this can increase healthcare costs and require skilled staff. The risk of bias was moderate to high in several domains due to unclear risks making it difficult to ascertain trial quality, which is another challenge.

## Conclusion

Regardless of possible limitations, the present meta-analysis indicates that perioperative glucose control using an intensive regimen is beneficial in decreasing the incidence of SSIs in some patient populations, and surgeries depending on the timing of administration and should be considered. The use of intensive glucose lowering strategies should be carried out with regard to current surgical practices and procedures and regarding the patient status with adequate safety monitoring.

## Data Availability Statement

The original contributions presented in the study are included in the article/supplementary material, further inquiries can be directed to the corresponding author/s.

## Ethics Statement

All procedures performed in the study were in accordance with the institutional and/or national research committee's standards and with the 1964 Helsinki declaration and its later amendments or comparable ethical standards.

## Author Contributions

JL and QL derived a concept and designed this study. YH analyzed data and drafting of this manuscript. SZ collected the data and helped in data analysis. XB and SR: proofreading and final editing along with guarantor of the manuscript. All authors read and approved the final version of the manuscript.

## Funding

Scientific research topics of Sichuan Institute of Health Information (2019040) Construction and Application of Intelligent Learning Adverse Event Reporting System Based on Clinical Knowledge Base.

## Conflict of Interest

The authors declare that the research was conducted in the absence of any commercial or financial relationships that could be construed as a potential conflict of interest.

## Publisher's Note

All claims expressed in this article are solely those of the authors and do not necessarily represent those of their affiliated organizations, or those of the publisher, the editors and the reviewers. Any product that may be evaluated in this article, or claim that may be made by its manufacturer, is not guaranteed or endorsed by the publisher.
