# Corrigendum: Glycemic Control Regimens in the Prevention of Surgical Site Infections: A Meta-Analysis of Randomized Clinical Trials

**DOI:** 10.3389/fsurg.2022.912295

**Published:** 2022-06-17

**Authors:** Jing Lai, Qihong Li, Ying He, Shiyue Zou, Xiaodong Bai, Sanjay Rastogi

**Affiliations:** ^1^ Department of Nursing, The First People's Hospital of Longquanyi District, Chengdu, China; ^2^ Department of Internal Medicine, Yantai Qishan Hospital, Yantai, China; ^3^ Department of Science and Teaching, The First People's Hospital of Longquanyi District, Chengdu, China; ^4^ Department of Endocrinology, The First People's Hospital of Longquanyi District, Chengdu, China; ^5^ Department of Outpatient, China Medical University, Shenyang, China; ^6^ Department of OMFS, Regional Dental College, Guwahati, Assam, India

**Keywords:** surgical site infections, glycemic control, laparoscopic surgeries, neurosurgeries, general surgery

**A Corrigendum on**

**Glycemic Control Regimens in the Prevention of Surgical Site Infections: A Meta-Analysis of Randomized Clinical Trials**

*by Lai, J., Li, Q., He, Y., Zou, S., Bai, X., and Rastogi, S. (2022). Front. Surg. 9:855409. doi: 10.3389/fsurg.2022.855409*

**Incorrect Affiliation**

In the published article, there was an error in affiliation ** Jing Lai^1†^, Qihong Li^2†^, Ying He^1^, Shiyue Zou^1^, Xiaodong Bai^3^* and Sanjay Rastogi^4^**. Instead of “**^1^Department of Nursing, The First People’s Hospital of Longquanyi District, Chengdu, China,

***^2^Department of Internal Medicine, Yantai Qishan Hospital, Yantai, China,***

***^3^Department of Science and Teaching, The First People’s Hospital of Longquanyi District, Chengdu, China,***

***^4^Department of Endocrinology, The First People’s Hospital of Longquanyi District,***

Chengdu, China**”,

It should be “**

1. Jing Lai

Department of Nursing, The First People’s Hospital of Longquanyi District, Chengdu, Sichuan, 610100, China

2. Qihang Li

Department of Internal Medicine, Yantai Qishan Hospital, Yantai, Shandong, 264001, China

3. Ying He

Department of Science and Teaching, The First People’s Hospital of Longquanyi District, Chengdu, Sichuan, 610100, China.

4. Shiyue Zhou

Department of Endocrinology, The First People’s Hospital of Longquanyi District, Chengdu, Sichuan, 610100, China.

5. Xiaodong Bai

Department of Outpatient, China Medical University, Shenyang, Liaoning, 110122, China.

6. Sanjay Rastogi

Department of OMFS, Regional Dental College, Guwahati, Assam, India.

**”. The authors apologize for this error and state that this does not change the scientific conclusions of the article in any way. The original article has been updated.

